# Secretion of placental peptide hormones: functions and trafficking

**DOI:** 10.3389/fendo.2025.1584303

**Published:** 2025-06-12

**Authors:** Sadia M. Ahmadi, Maira L. Perez, Carlos M. Guardia

**Affiliations:** Placental Cell Biology Group, National Institute of Environmental Health Sciences, National Institutes of Health, Durham, NC, United States

**Keywords:** human placenta, peptide hormones, secretion, pregnancy, cell biology

## Abstract

The placenta is a dynamic endocrine organ that plays a crucial role in fetal development by secreting a diverse array of peptide hormones that regulate maternal and fetal physiology. These hormones, including human chorionic gonadotropin (hCG), human placental lactogen (hPL), and placental growth hormone (hPGH), among others, are essential for pregnancy maintenance, fetal growth, and metabolic adaptation. Dysregulation of the secretory machinery and the levels of these hormones in circulation is associated with a myriad of pregnancy-related disorders. Despite their significance, the mechanisms governing their intracellular trafficking and secretion remain incompletely understood. This review synthesizes current knowledge on the secretion pathways of placental hormones, highlighting the interplay between constitutive and regulated secretion, and the challenges in defining these mechanisms due to the unique structure of the syncytiotrophoblast. We also discuss how emerging technologies, such as 2D and 3D placental models and advanced protein trafficking assays, can provide deeper insights into the regulation of placental hormone secretion. Understanding these processes will not only enhance our knowledge of placental biology but also provide new avenues for diagnosing and treating pregnancy-related disorders.

## Introduction

The placenta is credited for its importance in mammalian *in utero* development. As the first and largest fetal organ to develop, the placenta is the medium through which oxygen and nutrients are provided to the embryo alongside other metabolic and endocrinologic functions (1–4). Disorders in human placental development can have consequences in the health of the fetus and pregnant host that are lifelong. Common diseases during pregnancy, such as preeclampsia and intrauterine growth restriction, are attributed to defects in placental development and secretion (5, 6). The effects of fetal development on the predisposition to health complications in adulthood have already been proposed, according to the fetal origins hypothesis (7).

In order to assure the proper execution of the fetal development program, the placenta secretes a variety of hormones throughout the entire pregnancy. These hormones are mostly proteins and are produced and secreted through the secretory pathway (8–10). A compartmentalized cell maintains correct folding, processing, and delivery of all proteins entering and exiting the secretory cells dynamically. Placental hormones are mostly produced by a special cellular structure called syncytiotrophoblast. This multinucleated epithelium layer emerges in direct contact with the blood of the pregnant host as a result of regulated cell-cell fusion (11). The complexity of this cellular scenario makes it difficult to study intracellular trafficking events, including hormone secretion. Accordingly, dysregulation of such pathways has significant effects on placenta function and development.

Despite its long-lasting contribution to health and disease, both within and beyond the scope of reproduction, the human placenta remains largely overlooked in scientific research and its complexities are not fully understood. Ethical and practical implications for achieving a human placental model creates additional obstacles in research. The purpose of this review is to outline key components of placental development with a focus on protein trafficking based on published work. The compilation of existing knowledge is short of extensive, given the ongoing gaps in placenta research. We also encourage an open discussion on the future of placental research through emerging technologies, such as 2D and 3D lab-generated models, that can illuminate placental development and offer new tools to interrogate cellular mechanisms in greater detail.

## Placenta development

**Figure 1:**
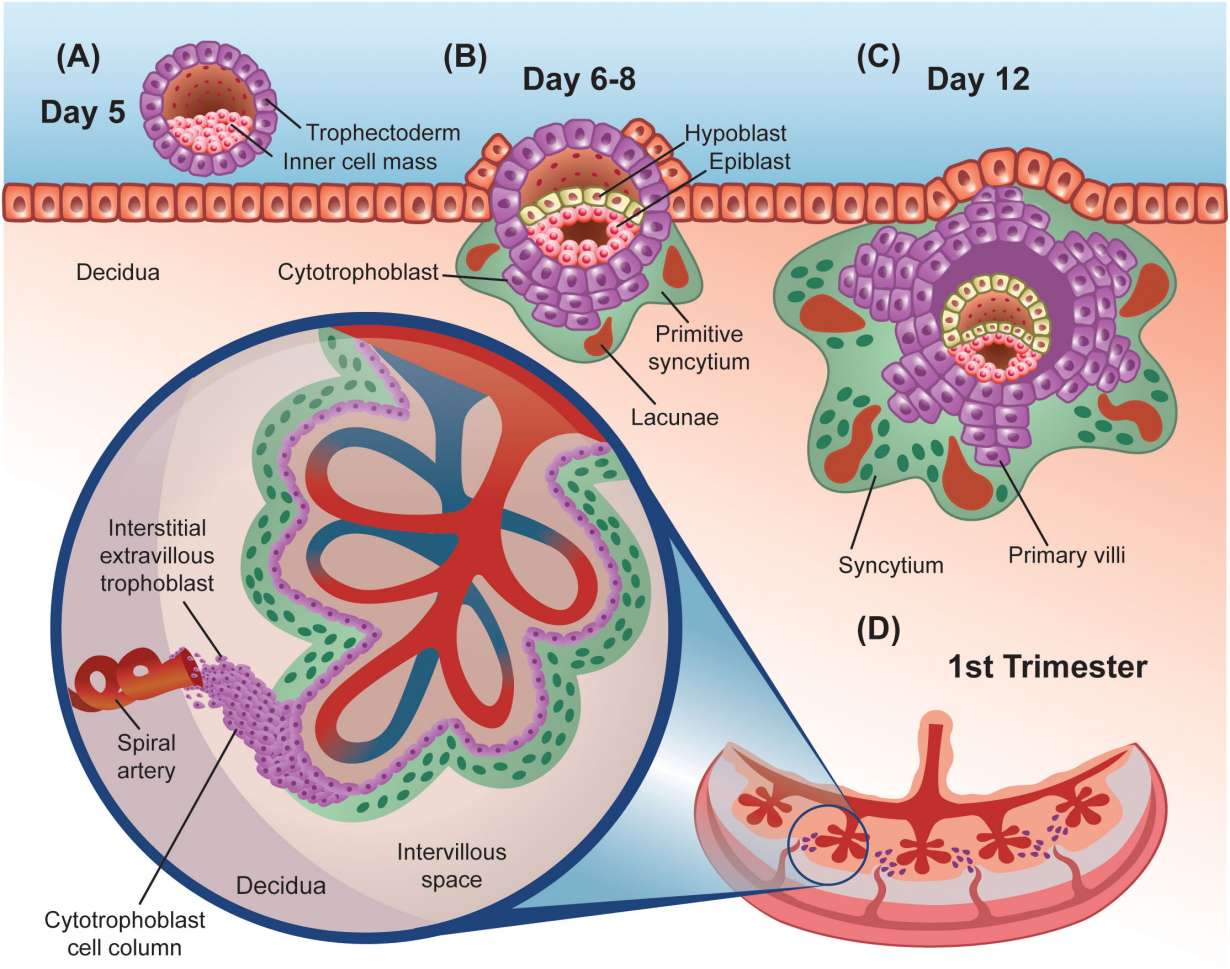
Graphic representation of early stages of human placenta development. **(A)** Preimplantation embryo, showing the external trophectoderm cell layer that protects the inner cell mass or embryo proper, in close contact with a layer of uterine epithelial cells that rest on top of the decidua. **(B, C)** Early post-implantation embryo, showing the primitive syncytium surrounded by lacunae and primary villi made out of cytotrophoblasts. **(D)** First trimester fully developed placenta. Inset shows the detailed cellular architecture of an anchoring villus. The cytotrophoblast cell column keeps this villus attached to the decidua and provides the interstitial extravillous trophoblasts that invade nearby spiral arteries, plugging them first and remodeling them later in pregnancy.

The origins of the human placenta can be traced to the trophectoderm, visible around day 5 post-fertilization. The trophectoderm is the external layer of the blastocyst and has a region in contact with the inner cell mass (12) (**Figure 1A**). This polar side of the trophectoderm, proximal to the inner cell mass, adheres to the uterine epithelium during embryo implantation. The trophectoderm subsequently transforms into the trophoblast and initiates the implantation process around day 6 post-fertilization (13).

Trophoblastic cells proliferate throughout the invasion of the uterine epithelium, forming a dual layer structure. The inner layer is comprised of mono-nucleated cytotrophoblast cells (CTB) that are initially shielded from the host tissue by a primitive, external syncytiotrophoblast (STB) (**Figure 1B**). The STB is a continuous multi-nucleated structure that forms from the fusion of neighboring CTBs (11). Across the surface of the STB, microvilli function as a region of contact between the placenta and the pregnant host and continue to expand until term (14). More broadly and later in development, the STB is regarded as the primary site for nutrient and gas exchange and noted for its endocrine function (15, 16).

The lacunar stage occurs between days 8 and 13 post-fertilization and is characterized by the appearance of fluid-filled masses within the primitive STB that grow to form lacunae (**Figure 1C**). Trabeculae, which are bands of the STB, separate the lacunae from one another (12, 17). The formation of the lacunar system divides the trophoblast layer into three sublayers: a primary chorionic plate, the lacunar system, and the trophoblastic shell (18). The STB continues to invade the uterus into the endometrium which transforms into decidual tissue and completely engulfs the blastocyst by day 12 post-conception (19, 20). Specifically, the decidual tissue beneath the blastocyst, and subsequently placenta, is termed the decidua basalis (12). Decidual cells are derived from the proliferation and differentiation of endometrial stromal cells during early implantation (18). Trophoblastic proliferation exhibits higher density on the side of the blastocyst that implanted (implantation pole), therefore this site will eventually give rise to the placenta (18).

Around day 13, the underlying CTB cells proliferate to form projections that extend through the STB into the trabeculae and invade the lacunar system (**Figure 1C**). This marks the start of the villous stage, happening between days 13 and 28 post-fertilization. Proliferation and branching of the trophoblast lead to the formation of primary villous trees, with the lacunae functioning as the intervillous space. The invasive nature of the trophoblast also causes the release of erythrocytes from maternal capillaries into the lacunae (18). Mesenchymal cells emerge around day 14 and proliferate along the interior lining of the cytotrophoblast cells (18). CTB cells continue to grow to form a continuous, shell structure around the villi that separate it from the decidua around day 15, with some CTB cells invading the decidua as extravillous trophoblasts (EVTs).

Mesenchymal cells from the embryo grow through the villous core, invading the primary villi, to form secondary villi (21). Between days 18 and 20, fetal capillaries emerge in the mesenchyme, resulting in the establishment of tertiary villi upon the cross sectioning of fetal capillaries in the villous stroma (22, 23) (**Figure 1D**). Fetal circulation will be separated from maternal blood via the placental barrier which consists of a continuous STB lining the intervillous space, a layer of villous CTB cells, a basal lamina (made of mostly laminin, collagen, and fibronectin), connective tissue, and fetal endothelium (18). At the start of the fifth week, the framework of the placenta is established. Vascular remodeling is also apparent near the end of the first trimester.

In summary, throughout the first month of pregnancy, the intricate process of placental formation unfolds, with the CTBs serving as a perpetual stem cell constantly regenerating the STB and monitoring fetal needs given the proximity to the fetal circulation. The STB responds to their environment via developmentally timed endocytosis and secretion of a myriad of hormones, and the EVTs are active in remodeling and regulating host blood flow. Altogether, these three main trophoblast cell types orchestrate the formation and function of the placenta for the entire pregnancy period.

## Placental protein hormones

**Figure 2:**
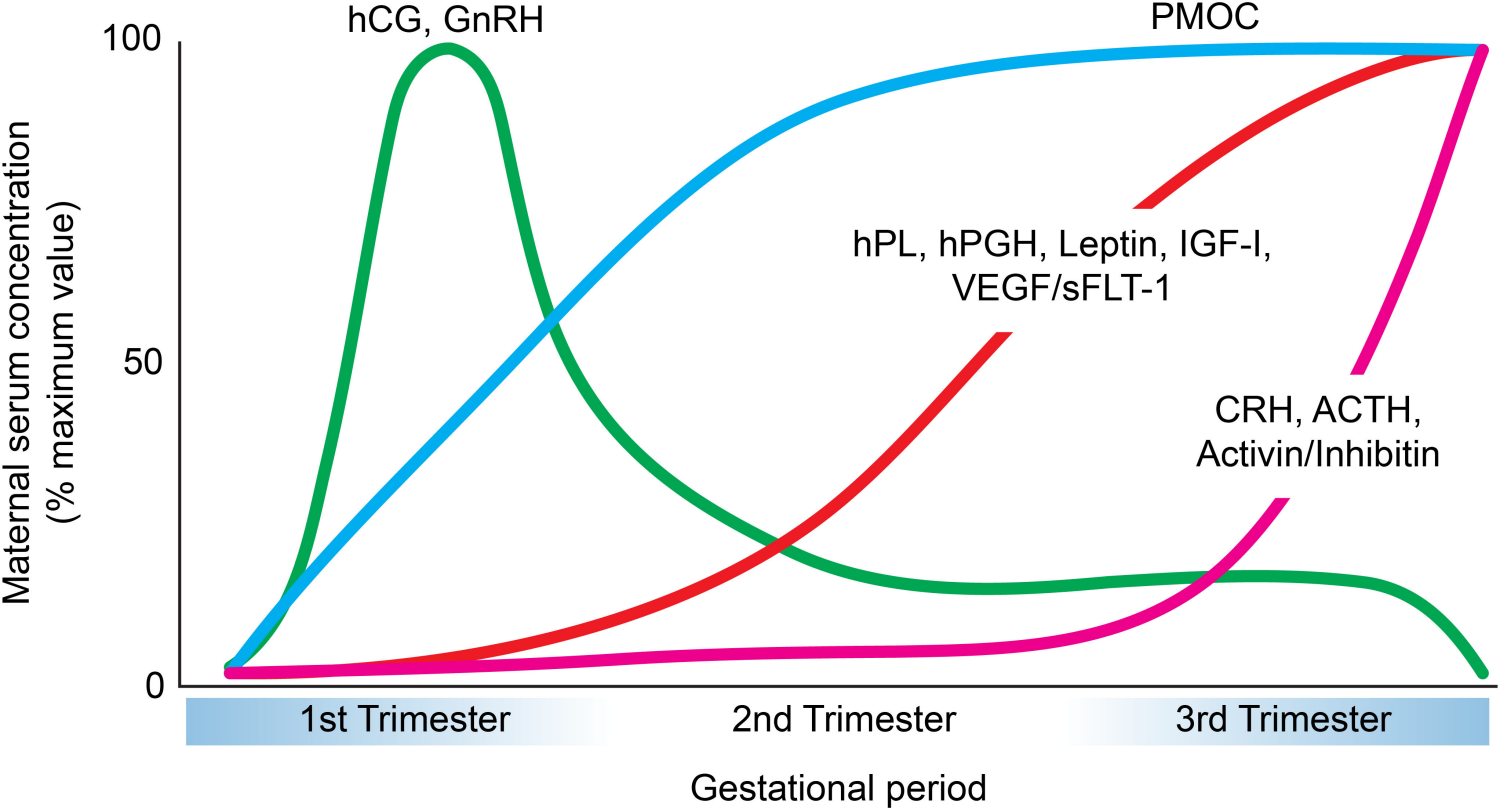
Temporal patterns of major placental peptide hormone secretion across pregnancy. Graphical representation of the maternal serum concentrations (expressed as a percentage of maximum value) of key placental hormones during gestation. Human chorionic gonadotropin (hCG) and gonadotropin-releasing hormone (GnRH) peak during the first trimester and decline thereafter (104, 250). Placental growth hormone (hPGH), human placental lactogen (hPL), leptin, insulin-like growth factor-I (IGF-I), and vascular endothelial growth factor/soluble fms-like tyrosine kinase-1 (VEGF/sFLT-1) progressively increase during the second and third trimesters (250–256). Proopiomelanocortin (PMOC) shows a steady rise throughout pregnancy that plateus during the second trimester (257). Finally, corticotropin-releasing hormone (CRH), adrenocorticotropic hormone (ACTH), and activin/inhibin demonstrate a marked elevation toward late gestation (258–261).

More than a passive physical barrier, the placenta is a highly active endocrine organ. Placental cells release large quantities of hormones in the host bloodstream throughout pregnancy that are vital for gestational success (**Figure 2**). Placental hormones are predominantly made by the STB, although CTBs and EVTs are also observed to have a role in hormone production (24, 25). The trafficking of these placental hormones can be an important indicator of pregnancy-related diseases. This section explores the major protein hormones secreted by the placenta, including their cellular origin, functions, and mechanisms in particular physiological pathways (**Table 1**).

**Table 1:** Summary of the different placental hormones described in this review.

Hormone	Placental origin	Target receptor	Functions	Associated pathologies
hCG^(a)^ 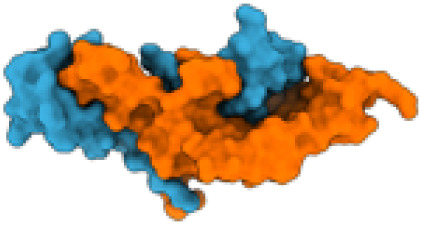	STB EVT	LH-hCG	↑ progesterone synthesis ↑ CTB differentiation (syncytialization) ↑ trophoblast invasion ↑ VEGF production	Pregnancy loss Preeclampsia
hPL^(b)^ 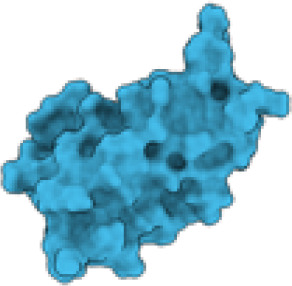	STB EVT	PRLR & GRH	↑ glucose uptake, metabolism, storage ↑ lipolysis ↑ pancreatic islets ↑ insulin secretion and resistance ↑ fetal anabolism	Low fetal birth weight Pregnancy loss Perinatal maternal mood
hPGH^(c)^ 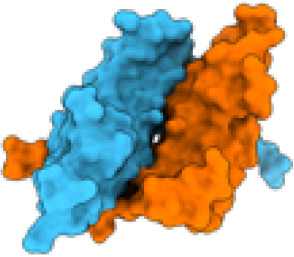	STB EVT	GRH	↑ IGF-I synthesis ↑ insulin resistance ↑ gluconeogenesis ↑ lipolysis ↑ trophoblast invasion	Low fetal weight Preeclampsia?
Leptin^(d)^ 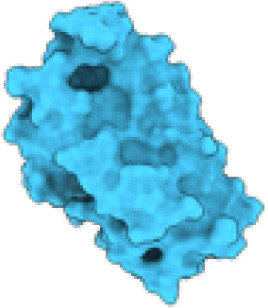	STB EVT	LepRb	↑ hCG secretion ↓ progesterone synthesis ↑ CTB proliferation, ↓ CTB apoptosis ↑ trophoblast invasion ↑ placental angiogenesis ↑ lipolysis ↓ placental triglyceride and cholesterol levels ↑ amino acid transport	Pregnancy loss Preeclampsia Gestational diabetes Intrauterine fetal growth restriction
GnRHs^(e)^ 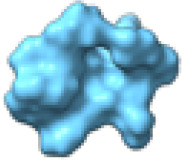	**GnRH-I**STBCTBEVT**GnRH-II**CTBEVT	Type 1 GnRHR	↑ hCG secretion ↑ FSH and LH secretion ↑ trophoblast invasion	Pregnancy loss Still birth Low fetal birth weight
Activin & Inhibin^(f)^ 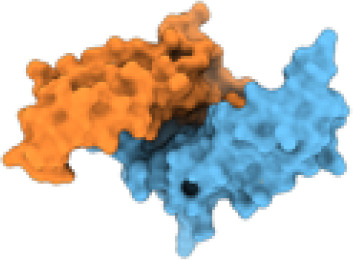	STB CTB	ActRIIA & ActRIIB	**Activin** ↑ FSH secretion ↑ CTB differentiation ↑ trophoblast invasion ↑ GnRH activity ↑ hCG secretion **Inhibin** ↓ FSH secretion ↓ CTB differentiation ↓ trophoblast invasion ↓ GnRH activity ↓ hCG secretion	Pre-term labor Preeclampsia Gestational diabetes
CRH^(g)^ 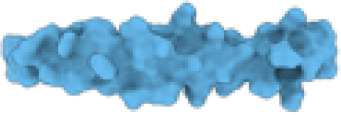	STB	CRH-R1 & CRH-R2	↑ POMC and ACTH synthesis ↑ prostaglandin secretion ↑ fetal steroidogenesis ↑ placental vasodilation	Pre-term labor Hypertension Impaired uterine artery blood flow Preeclampsia
POMC	STB	–	Precursor to ACTH	–
IGFs^(h)^ 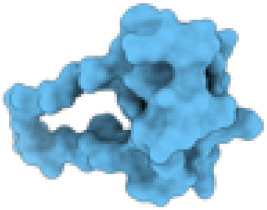	**IGF-I**STBCTB**IGF-II**CTBEVT	IGF-IR IGF-IIR (IGF-II) IR (IGF-II)	↑ trophoblast invasion ↑ CTB proliferation ↑ amino acid transport	Impaired placental growth Fetal growth restriction Preeclampsia Gestational diabetes
VEGF^(i)^ 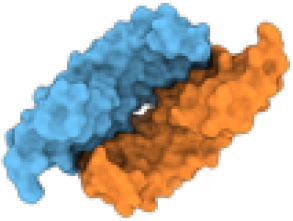	STB	KDR & FLT-1	↑ placental vasodilation ↑ vascular permeability ↓ epithelial cell apoptosis ↑ spiral artery remodeling	Preeclampsia

The 3D structure of each hormone has been included in the first column, according to the experimentally determined structures (PDBIDs): (a) 1HRP; (b) 1Z7C; (c) 1FZV; (d) 8K6Z; (e) 4D5M; (f) 2ARV (Activin A homodimer); (g) 1GO9; (h) 6PYH (IGF-I); and (i) 1VPF (VEGFA). Figures were created using ChimeraX 1.7 software (249).

### hCG

Human chorionic gonadotropin (hCG) is a heterodimeric glycoprotein with two subunits, hCG-α and hCG-β. This hormone is primarily generated by the STB and may possibly be synthesized by EVTs as well (26). The levels of hCG rapidly increase during the first trimester, with peak concentration near week 10 of pregnancy. Between 12 to 16 weeks, however, hCG levels gradually decline to a fifth of the peak concentration until term (27) (**Figure 2**).

This hormone binds to a G-protein coupled receptor that is also receptive to luteinizing hormone (LH). The LH-hCG receptor is expressed predominantly in gonadal and uterine cells (28), as well as placental STB, CTBs and EVTs (29, 30). Binding of hCG to its LH-hCG receptor leads to the activation of adenylyl cyclase and phospholipase C. Stimulation of adenylyl cyclase results in higher intracellular concentrations of cyclic adenosine monophosphate (cAMP), whereas phospholipase C subsequently generates inositol phosphates and increases intracellular calcium levels (31).

The recognition and maintenance of pregnancy is mediated by hCG. Binding of hCG to LH receptors on ovarian corpus luteum (CL) cells prevents luteolysis and prolongs CL function. In doing so, hCG promotes progesterone production via the CL that is necessary for averting menstruation and establishing pregnancy (32). It is also proposed that hCG facilitates the fusion of trophoblast cells, particularly the differentiation of CTBs into the STB. This is made possible by the increase in cAMP which initiates the protein kinase A pathway and activates downstream proteins, such as glial cells missing transcripton factor 1 (GCM1) (33). Targets of the GCM1 protein include the endogenous retroviral protein syncytin-1, which has direct involvement in CTB fusion (34). Notably, there is an upregulation of placental proteins specific for the STB, such as the beta-subunit of hCG (hCG-β) after syncytialization (33). The hCG hormone also enhances trophoblast invasion through increasing CTB secretion of matrix metalloproteinases MMP-2 and MMP-9 and decreasing the levels of their TIMP inhibitors TIMP-1, TIMP-2, and TIMP-3 (35). These effects are achieved by the hCG-mediated activation of ERK and AKT signaling pathways (36). Additionally, hyperglycosylated hCG, an hCG variant, binds and antagonizes TFGβ receptors which allows for the tumor-like invasion of trophoblast cells into the uterus (37). The secretion of hCG is enhanced by the cAMP-PKA and ERK pathways alongside other hormones like GnRH, leptin, and activin (38–42). Hormones such as inhibin and progesterone are noted for their antagonistic effects on hCG secretion (42, 43). Other observed functions characterize hCG as an angiogenic factor contributing to placental and uterine vascularization (44–46). Additionally, hCG plays a role in promoting vascular endothelial growth factor (VEGF) production by the STB, facilitating umbilical cord growth (47–49), and promoting fetal organ development, a suggestion made after hCG/LH receptors were found in fetal organs that are not present in adult organs (37). Given the importance of hCG, defects in expression of this hormone can have dire outcomes like miscarriage and preeclampsia (50–52).

### hPL

Human placental lactogen (hPL) is a polypeptide hormone primarily generated by the STB, but it can also be synthesized by EVTs (25). This hormone is initially detected 5–10 days after implantation and becomes visible in host plasma near the 6^th^ week of pregnancy. The levels of hPL increase consistently and reach a peak at 30 weeks (**Figure 2**). Interestingly, hPL concentration is positively correlated with the placental mass and number of growing fetuses, suggesting a role for hPL on placental and fetal growth (53).

This hormone binds to the human prolactin receptor (PRLR) as well as the human growth hormone receptor (GHR), although with lower affinity to the latter (54). Prolactin receptors are widely expressed across various tissues, including the mammary gland, ovary, and pancreas (55, 56). The functions of hPL pertain mostly to carbohydrate and lipid metabolism. It has been observed that hPL reduces insulin sensitivity of the host during pregnancy, while promoting glucose uptake, oxidation, and storage as glycogen (53). Additionally, hPL increases the rates of lipolysis *in vitro* as well as the plasma concentrations of fatty acids, ketones, and glycerol *in vivo*, all of which can support fetal development (53). Furthermore, hPL contributes to the formation of pancreatic islets and upregulates insulin secretion in the host (57, 58). Studies also suggest a role for hPL in breast epithelial cell proliferation of the host, presumably in preparation for lactation (59). With respect to the fetus, hPL is noted to encourage fetal anabolism by stimulating DNA synthesis, amino acid uptake, and IGF-I production (60). Despite its relatively lower concentration in the fetal circulation compared to the host, hPL is speculated to play a part in fetal pancreatic development, although it is unknown whether this hormone regulates fetal islets similar to maternal islets (53, 61, 62). Abnormalities in hPL secretion are associated with low fetal birth weight, perinatal maternal mood, and pregnancy loss (63–65). Molecules that stimulate hPL release include cAMP, insulin, and growth hormone releasing hormone (66–68). Somatostatin has been associated with inhibitory effects on hPL secretion due to its expression levels during pregnancy that are inverse to that of hPL (69).

### hPGH

Human placental growth hormone (hPGH) is a polypeptide made by the STB, however it can also be produced by EVTs (70). This hormone is distinguished from its pituitary human growth hormone (hGH) counterpart (secreted by the somatotropic cells of the anterior pituitary gland) based on a difference of 13 amino acids (71). It appears that hPGH emerges in host circulation around the second half of pregnancy and begins to dominate over pituitary growth hormone, continuing to rise until term (72) (**Figure 2**).

This hormone binds to the hGH receptor (GHR), abundantly expressed in liver cells, as well as the prolactin receptor (54, 73). The function of hPGH has been linked to promoting maternal IGF-I synthesis and insulin resistance (74, 75). Moreover, hPGH exerts metabolic effects through enhancing gluconeogenesis and lipolysis, thus allowing for higher nutrient availability to the fetoplacental unit (71). The combined effects of hPGH on IGF-I production and host metabolism are correlated with fetal development (76). Additionally, hPGH increases the invasion of EVTs, directly contributing to the growth of the placenta (70). Reductions in hPGH expression may hinder IGF-I production and indirectly cause low fetal birth weight, whereas association of hPGH levels to preeclampsia is conflicting (77). The secretion of the hPGH hormone is promoted by cAMP and inhibited by glucose, leptin, and insulin (71, 78).

### Leptin

Leptin is a peptide hormone largely produced by the STB and EVTs (79, 80). The concentration of leptin increases dramatically during the first and second trimester, peaking near week 28, and then declines rapidly after parturition (81, 82) (**Figure 2**).

Leptin binds to the long-form Leptin receptor LepRb, highly expressed in cells of the hypothalamus and placenta, and activates the ERK and JAK2-STAT5 pathways (83, 84). This hormone increases the secretion of hCG and simultaneously inhibits the synthesis of progesterone and hPGH (41, 78). Leptin also enhances CTB proliferation through upregulating cyclin-D1 and inhibiting apoptosis to advance cell cycle progression to the G2/M stage (85). Furthermore, leptin promotes trophoblast invasion by inducing the expression of MMP-2 and MMP-9 (80). Leptin function has also been linked to placental angiogenesis, leading to the formation of capillary-like tubes *in vitro (* 86). Additionally, leptin was found to have catabolic effects on the host, including lipolysis and the reduction of placental triglyceride and cholesterol levels (87). It has also been demonstrated that leptin increases host-derived amino acid transport, thereby contributing to the growth of the fetus (88). Dysregulation of leptin expression is related to pathologies such as gestational diabetes, recurrent miscarriage, preeclampsia, and intrauterine fetal growth restriction (89–94) Leptin production is enhanced by the cAMP, ERK, and PKA/PKC pathways (95, 96). Molecules such as hCG and insulin also promote leptin secretion (97, 98). Hormones such as hPL and progesterone antagonize the release of leptin (99).

### GnRH

Gonadotropin hormone-releasing hormone (GnRH) is secreted in two forms by the placenta, GnRH-I and GnRH-II. Placental peptide GnRH-I is immunologically and biochemically equivalent to that of the hypothalamus, with the exception of differences in the 5’-untranslated region of the gene (100, 101). GnRH-I is produced by all the trophoblasts in the placenta. On the other hand, GnRH-II is expressed by CTBs and EVTs. GnRH-I and GnRH-II are both identified in first trimester placentas, although only GnRH-I is detected at term (102) (**Figure 2**).

The mechanisms of chorionic GnRH are yet to be clearly defined. Previous studies report that placental GnRH stimulates hCG secretion from the STB after binding to the type 1 GnRH receptor localized to the STB and CTBs (103, 104). It has been proposed that GnRH-stimulated hCG secretion is a receptor-mediated process (105). Parallel to hCG levels, placental GnRH receptors were observed to peak at week 9 of gestation, prior to declining between weeks 12–20 and later becoming undetectable at term (106). In addition, chorionic GnRH contributes to trophoblast invasion through regulation of MMP-2 and MMP-9 in primary EVTs (107, 108). Evidently, GnRH function corresponds to the establishment and maintenance of pregnancy and disruption of placental GnRH expression or receptor activity can lead to unfavorable pregnancy outcomes including pregnancy loss, stillbirth, and low fetal birth weight (109–111). The secretion of chorionic GnRH is upregulated by molecules such as cAMP and activin (112).

### Activin/Inhibin

Activins and inhibins are dimeric glycoproteins that are characterized for their regulatory nature. More specifically, inhibins are heterodimers with α and β subunits, and prevent the release of FSH from the pituitary system (113). Activins can be homo or heterodimers of β-inhibin and exhibit opposing effects for that of inhibin by functioning as a stimulant for the release of FSH (113, 114). Activin A and Inhibins A and B are predominantly synthesized by the STB, although they can also be generated by the CTB (115, 116). These molecules are observed to have rising concentrations during pregnancy (117) (**Figure 2**).

Activin A binds to type II activin receptors, ActRIIA and ActRIIB, located mainly on the STB of the placenta, among other cells in the pituitary, hypothalamus, or gonads (118–121). Activins facilitate CTB differentiation into the STB and EVT and also promote trophoblast invasion (122–124). With its shared β-subunit, inhibins competitively bind to activin receptors and serve as regulators for activin (125). Inhibin reverses the effects of activin by suppressing GnRH activity, which then results in a decrease in hCG production (42). Disruptions in the secretion of activins and inhibins have been linked to pathologies such as spontaneous or pre-term labor as well as preeclampsia and gestational diabetes (126–129).

Inhibin secretion is promoted by molecules such as GnRH, hCG, and cAMP (130). The production of inhibin is downregulated by Activin A through a feedback loop (131). The release of activin is upregulated by CRH (132).

### CRH

Corticotropin-releasing hormone (CRH) is a peptide generated mainly by the STB (133, 134). Chorionic CRH is identical to CRH produced in the hypothalamus and is detected in low levels between weeks 7-19, with rising concentrations during weeks 35–40 of gestation (135–138) (**Figure 2**). Placental CRH binds to receptors CRH-R1 and CRH-R2, located primarily in the pituitary and central nervous system as well as placental and fetal membranes (139, 140). Competition with CRH binding protein (CRH-BP), which has greater affinity to the CRH receptor, renders CRH in host circulation as inactive for most of gestation. CRH-BP levels decrease in the final weeks of pregnancy and coincides with the increase in CRH bioavailability and activity, marking CRH as a signal for parturition (141). The functions of CRH encompass increasing intracellular cAMP and enhancing the synthesis of both proopiomelanocortin (POMC) and its derivative adrenocorticotropic hormone (ACTH) in the placenta (142, 143). Additionally, CRH stimulates placental secretion of prostaglandins, possibly contributing to myometrial contractions at term (144). Moreover, CRH facilitates steroid production by fetal adrenals, which may further incite parturition (145).

Urocortins are a subclass of CRH peptides that are primarily made by the STB and, to a lesser extent, CTBs (146). Urocortins bind to CRH receptors and play a role in placental vasodilation in addition to shared effects of increasing cAMP concentrations and promoting POMC (see below) and ACTH secretion by trophoblast cells (147–149).

Improper release of CRH has been associated with adverse pregnancy outcomes such as pre-term labor, hypertension, impaired uterine artery blood flow, and preeclampsia (150–153). CRH secretion is upregulated by glucocorticoids and prostaglandins and obstructed by progesterone (144, 154).

### POMC

Proopiomelanocortin (POMC) is a glycoprotein precursor to ACTH with proposed synthesis by the STB (155, 156). Although undetected in normal subjects, POMC is readily identified by the third month of gestation for pregnant individuals (**Figure 2**). This is attributed to differences in the processing of POMC between the pituitary and the placenta, as the latter secretes bulk quantities of the intact, uncleaved hormone (157). POMC levels gradually increase throughout pregnancy and are positively correlated with CRH concentration, however demonstrate no relationship with ACTH or cortisol levels (157). The functional significance of placental ACTH and other POMC-derivatives remains largely unspecified.

### IGF-I and IGF-II

Insulin-like growth factors IGF-I and IGF-II are produced by the placenta and play a critical role in fetal development (158). IGF-I is secreted by the STB and CTBs throughout pregnancy (**Figure 2**). On the other hand, IGF-II is generated by CTBs and EVTs during the first trimester (159).

These peptides bind to tyrosine kinase receptor IGF-IR present in all placental cell types, while IGF-II can also bind to the IGF-II and insulin receptors (160, 161). IGFs stimulate trophoblast invasion and are also involved in the proliferation of CTBs via the PI3K and MAPK pathways (162–164). Moreover, IGFs contribute to fetal development by facilitating amino acid transport across the placenta (165). These molecules are regulated by IGF binding proteins that limit their availability to interact with IGF receptors (166). Alterations to placental IGF expression demonstrate consequences of restricted placental and fetal growth, and are implicated to have a pathological role in preeclampsia and gestational diabetes (158, 166, 167).

### VEGF

Vascular endothelial growth factors (VEGFs) are a family of proteins that are characterized for their angiogenic properties. During gestation, VEGF-A and placental growth factor (PlGF) are secreted from the STB (168) (**Figure 2**). These molecules bind to placental tyrosine kinase receptors KDR and FLT-1, and are downregulated by association with the soluble form of FLT-1 (sFLT-1) (169). The functions of VEGFs are strongly linked to vascular endothelial cell proliferation and angiogenesis through effects of increased vasodilation and vascular permeability (170–172). Angiogenesis is further promoted via the antiapoptotic support VEGFs provide placental epithelial cells (173). VEGFs also influence the remodeling of host spiral arteries that is critical for gestational success (174, 175). Complications with VEGF/PlGF secretion can be indicative of pathologies like preeclampsia (176).

## How does secretion of placental peptide hormones happen?

**Figure 3:**
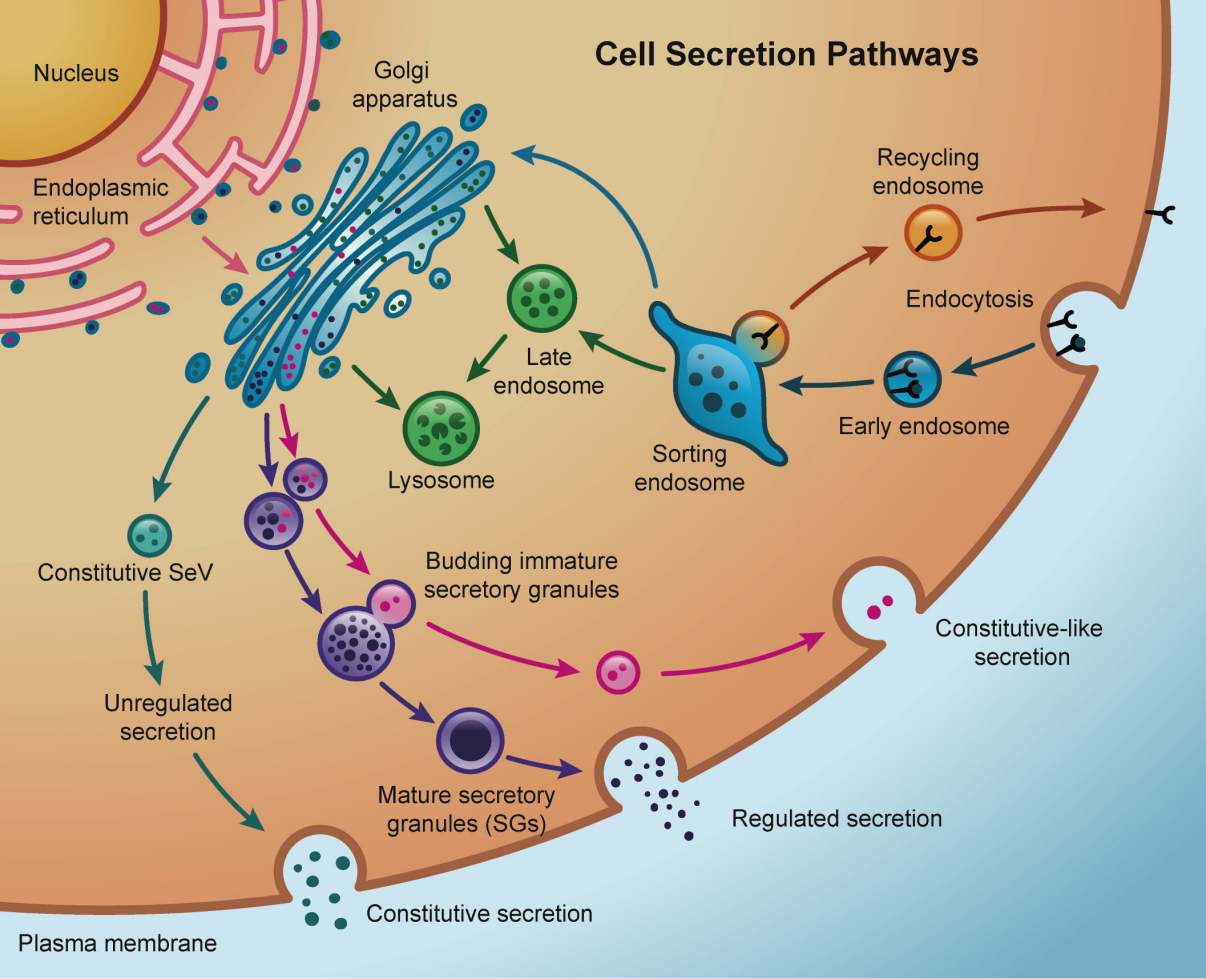
Schematic representation of the main secretion pathways in a cell. Active endocytosis and recycling through sorting endosomes allows the cell the control of cargo internalization and the reutilization of certain receptors depending on the intracellular demands. Cargo and receptors no longer required by the cell are degraded inside late endosomes and lysosomes, that receive lysosomal-specific degradative enzymes via direct trafficking from the Golgi or indirectly from the plasma membrane. Constitutive secretion brings Golgi newly synthesized in secretory vesicles (SeV) or recycled cargo directly back to the plasma membrane. In contrast, regulated secretion involves the packaging of cargo into highly-regulated secretory granules (SGs). Sorting of specific cargo from immature secretory granules constitutes constitutive-like secretion pathways.

Transport of secreted proteins in eukaryote cells requires specialized membrane and cytosolic machinery of the secretory and endo-lysosomal pathways (8–10) (**Figure 3**). Most of the conventional secretory proteins are synthesized in the endoplasmic reticulum (ER) lumen via N-terminal signal sequence recruitment of ribosomes. Here, proteins fold and some acquire complex glycans that are further processed once the protein is exported to the Golgi apparatus. From there, proteins are sorted and trafficked to the cell surface via membrane-bound vesicles or tubular carriers (177–179). This rapid delivery of cargo to the extracellular space is described as constitutive secretion and operates in all cell types (180). Specialized cells such as lactotrophs of the anterior pituitary gland or β-cells in the pancreas, for example, produce large amounts of specific proteins that are stored in granules (secretory granules – SGs) at high concentration before being exocytosed upon stimulation. The formation, maturation, and release of these SGs require very specific trafficking and signaling regulation (181–184). Regulated secretory proteins contained in SGs include hormones, neuropeptides, enzymes, and extracellular components of mucus.

Although presented as black-and-white alternative pathways, both constitutive and regulated secretion are not mutually exclusive, and a degree of overlapping could be observed in different systems (185, 186). For example, studies in acinar cells of the salivary glands (187) and pancreatic islets (188–191) have shown a constitutive-like pathway through endosomes where minor regulated secretion from immature SGs takes place under basal (i.e., no stimulus) conditions. Most of secreted proteins from salivary cells follow an exocrine, regulated pathway into the saliva but constitutive secretion is also observed (192, 193). Similarly, mucin secretion from goblet cells in the airways utilizes both constitutively and regulated secretion pathways (194). Additionally, lysosome-related organelles (LROs) are found and used to store and secrete proteins in many specialized cells different from exocrine cells (195–197). The biogenesis of many LROs involves common components of the endolysosomal and regulated secretory pathways, such as small GTPases, molecular motors, and sorting adaptor proteins and complexes (198).

The placenta, as a unique endocrine organ of temporary function, offers a particularly interesting system of study of protein secretion. As presented above, the placenta secretes a myriad of protein hormones and peptides. The most abundant ones (hCG and hPL) have high homology to pituitary hormones (hCG: 82% with LH (199); hPL: 85% with GH but 22% with hPRL (200)) which are shown to be secreted in SGs. Structurally, both hormones are very different from each other (**Table 1**), and they are secreted at different rates during pregnancy (37, 201) (**Figure 2**). How these two hormones are secreted by the placenta has been a controversial topic of discussion during early placental research.

Contemporary to the revolutionary discoveries of George Palade and colleagues by extensive use of ultrastructural characterization of the intracellular membranous system in specialized secretory cells (202), many researchers dug into similar approaches to uncover the localization of placental hormones. Initial studies using both first trimester and term placenta in resin embedded sections (203–205) showed a wide range of thin, smooth membrane-surrounded dense granules within or near the Golgi apparatus that were different from lipid droplets. Larger granules were observed near the apical face of the STB while smaller ones more dispersed around the cytosol, suggesting the former are originated from homotypic fusion between the latter. Although sparse and initially mistakenly as vesicles packed with steroid hormones, the existence of such SG-like structures was very evident. It was even suggested that the small SGs bud off the limiting membrane of the large vesicles in a mechanism that we would associate nowadays with constitutive-like biogenesis (206). A study using cryosections of human term placentas and HRP-conjugated antibodies to improve detection of hCG confirmed infrequent granules and a strong immunoreactivity in the plasma membrane of the STB (207), a stain that could not be observed later by others using similar approaches (208). The simultaneous establishment of the first human hormone-producing trophoblastic cell line (209), the choriocarcinoma BeWo cell line, allowed the interrogation of SG production and secretion *in vitro*. Results from investigations using this model were, however, not consistent with regulated secretion of hCG: long incubation times with secretagogues was required to stimulate secretion (210), ionophore treatment did not trigger exocytosis (211), incubation with high concentrations of K^+^ or cytoskeleton drugs did not affect secretion (212, 213), and insignificant amounts of the hormone was observed in stored form (214). Later, similar results were obtained in other choriocarcinoma cell lines (JAR and JEG-3) (215–217). However, more studies using intact chorionic villi and different immunolabeling approaches (218–221) followed up and confirmed the presence of plentiful large bodies resembling large SGs reactive for hCG. One study demonstrated that these abundant granules in first trimester placentas are rich in iron (222) suggesting the possibility of LRO-like characteristics.

Using the pioneer method of isolation of primary trophoblasts from midterm and term placenta by Hall et al. (223), Hochberg, Bick, and Perlman started exploring similar questions than the ones described above but for the secretion of hPL (68, 224–226). These studies agree with the previously mentioned reports for hCG secretion where a constitutive type of secretion is favored. Perhaps differently from the studies with hCG, these authors explicitly recognized the presence of a small fraction of hPL that could be secreted from a prestored pool (68). Using term placenta explants, it was initially shown that hPL does not follow a regulated secretion behavior when K^+^ and Ca^2+^ levels are manipulated (227), but such results were contradicted later (228, 229) suggesting then the possibility of a mixed mechanism of secretion. Not surprisingly, immunoelectron microscopy experiments using human placenta tissue showed robust hCG and hPL in small, medium, and large granules in the STB (230, 231), highlighting the differences between *ex vivo* tissue phenotypes and cells in culture. Conversely, systematic studies by the group of Boime and colleagues, demonstrated that although some of the hCG is stored in SGs and secreted by regulated mechanisms, the vast majority of the hormone is released constitutively (232). But similar studies for hPL (and the many other secreted placental hormones) are still lacking and extrapolation of these results might not be appropriate given the different structural characteristics to hCG, leading to still a larger open question: how is hormonal secretion truly regulated in the placenta?

## Conclusions and future directions

The placenta has the remarkable task of coordinating the secretion of numerous substances using a very specialized and unique cellular structure: the STB. Although poorly characterized at the cellular level, the STB is constantly replenished with cytosol, membranes, and nuclei from the underlying CTB layer, perhaps as a mechanism to cope with this intense secretory duty in a local-specific fashion. It is still a mystery how this giant multinucleated structure manages to secrete, at different developmental milestones, different hormones that control its own and both the maternal and fetal fates during the entire pregnancy. The placenta undergoes continuous, sex-specific changes throughout gestation in response to the dynamic maternal-fetal environment to support healthy fetal development. From implantation to parturition, the placenta continuously senses and adapts to maternal and fetal signals, acting as a hormonally active organ uniquely present during pregnancy.

Progress in understanding placental secretory pathways has been limited by restricted access to early gestation tissue, species differences between humans and model organisms, and the limitations of *in vitro* systems in replicating key physiological processes. We showed a clear example of the challenges faced by the field to study the intracellular trafficking of the main placental hormones: hCG and hPL. Through the research highlighted here, early and term placenta tissue are substantially different, not only in developmental terms but also in secretory capacities. The early placenta expresses high amounts of hCG while the term placenta favors the production of hPL (**Figure 2**). Both hormones have been found to be constitutively secreted, but a portion has been found in SG-like structures that we still have not fully characterized. While useful for research, trophoblast cell lines and primary cells often fail to fully replicate hormone synthesis, glycosylation, and trafficking due to limitations in their derivation and differentiation. Limited focus on the secretory mechanisms of other protein hormones in the literature has slowed progress in resolving key questions about placental hormone secretion.

With the advent of novel trophoblast models in 2D and 3D (233, 234) in combination with modern techniques for following protein trafficking inside the cells (for example: combining vesicle relocalization with spatial proteomics (235); photoactivation assays (236); probabilistic density maps (237); RUSH trafficking assays (238); transient CRISPR KO technology (239)) we have the opportunity to enter a new chapter in the interrogation of secretory pathways in the placenta. With these advancements, it is essential to evaluate both the strengths and limitations of emerging technologies in the study of placental hormone biology. Human trophoblast organoid models have marked a significant step forward, offering 3D systems that closely mimic placental morphology and endocrine function *in vitro (* 240–242). These cultures can secrete physiologically relevant levels of hormones and support long-term experimentation. However, they primarily model early pregnancy, lack full *in vivo* architectural complexity, including complete syncytialization and maternal–fetal interface features, and current gene editing tools remain underdeveloped in these systems. Notably, many of the hormones discussed in this review have yet to be investigated in these model systems, highlighting a critical gap in validation and mechanistic understanding within the field. Similarly, advanced live-cell imaging methods—such as confocal, super-resolution, and lattice light-sheet microscopy—enable real-time tracking of vesicle trafficking and hormone secretion at high resolution (243–246). While these techniques are powerful, their application to placental tissues will require extensive optimization and access to specialized equipment, especially in thicker or more heterogeneous samples. Taken together, each technology provides a unique perspective, but a truly comprehensive understanding of placental cell biology will require integrative approaches that combine spatial, temporal, and functional data across multiple platforms.

Fields studying other secretory organs have faced similar challenges of fitting all the phenotypes into one or another model of secretion (193, 247, 248) and galvanized into mixed models that contemplate intermediate or completely new alternatives. Nonetheless, whether in SGs, vesicles, or both, many questions about placental hormone trafficking remain open. What is the machinery that regulates sorting and secretion in the morphologically complex syncytiotrophoblast? Are all the peptide hormones sorted together to the plasma membrane or do they arrive in different carriers? Given the fetal-maternal interface structure of the placenta, how is the polarized sorting controlled? What about the rest of the neglected placental secretome? It is our wish that the next generation of cell biology tools applied to the placenta and trophoblast fields can stimulate the development of specialized approaches and accelerate the discovery in this particularly challenging but equally exciting organ.
